# Preparation of Surface-Active Hyperbranched-Polymer-Encapsulated Nanometal as a Highly Efficient Cracking Catalyst for In Situ Combustion of Heavy Oil

**DOI:** 10.3390/molecules28145328

**Published:** 2023-07-11

**Authors:** Ao Sun, Chenyang Yao, Lifeng Zhang, Yanmin Sun, Jun Nan, Houkai Teng, Jiazhong Zang, Lishan Zhou, Zhenzhong Fan, Qilei Tong

**Affiliations:** 1 Bohai Rim Energy Research Institute, Northeast Petroleum University, Daqing 163318, China; sunaonepu@hotmail.com (A.S.);; 2 Department of Chemistry, Zhejiang University, Hangzhou 310058, China; 22037068@zju.edu.cn; 3 CenerTech Tianjin Chemical Research and Design Institute Co., Ltd., Tianjin 300131, China; zlifeng@zju.edu.cn (L.Z.);; 4 Key Laboratory of Improving Oil Recovery by Ministry of Education, Northeast Petroleum University, Daqing 163318, China

**Keywords:** platinum nanoparticles, hyperbranched polymer, heavy oil, interfacial activity, thermal behavior

## Abstract

In situ combustion of heavy oil is currently the most suitable thermal method that meets energy consumption and carbon dioxide emission requirements for heavy oil recovery. The combustion catalyst needs to perform multiple roles for application; it should be capable of catalyzing heavy oil combustion at high temperatures, as well as be able to migrate in the geological formation for injection. In this work, a hyperbranched polymer composite nanometal fluid was used as the injection vector for a heavy oil in situ combustion catalyst, which enabled the catalyst to rapidly migrate to the surface of the oil phase in porous media and promoted heavy oil cracking deposition at high temperatures. Platinum (Pt) nanoparticles encapsulated with cetyl-hyperbranched poly(amide-amine) (CPAMAM), with high interfacial activity, were synthesized by a facile phase-transfer method; the resulting material is called Pt@CPAMAM. Pt@CPAMAM has good dispersion, and as an aqueous solution, it can reduce the interfacial tension between heavy oil and water. As a catalyst, it can improve the conversion rate during the pyrolysis of heavy oil in a nitrogen atmosphere. The catalyst structure designed in this study is closer to that exhibited in practical geological formation applications, making it a potential method for preparing catalysts for use in heavy oil in situ combustion to resolve the problem of catalyst migration in the geological formation.

## 1. Introduction

Heavy oil resources are globally abundant, but the difficulty and costs of the extraction of heavy oil are higher than those for ordinary crude oil. Oil in situ combustion technology is a highly efficient thermal recovery method for heavy oil; it involves continuously injecting heated oxygenated gas into the geological formation to ignite the crude oil and heat the geological formation. The displacement efficiency of oil in situ combustion can reach over 80% in laboratory simulations and 45–80% in field tests [1,2,3,4,5].

Compared with other thermal displacement technologies, using oil in situ combustion to release heat in the geological formation yields the highest energy savings and the lowest carbon emissions [6]. With increasing global carbon dioxide emissions, the environmental problems generated during the recovery process have attracted attention [7,8,9]. Therefore, improving the efficiency of mining and reducing the pollution of water bodies during the production process is essential [10,11,12].

During the in situ combustion process, a stable combustion leading edge is a key factor affecting the efficiency of the in situ combustion. At present, researchers divide the chemical reactions involving heavy oil during in situ combustion at temperature intervals into three stages: low-temperature oxidation, fuel deposition, and high-temperature oxidation [13,14,15]. The fuel deposition stage is the coupling zone of heavy oil oxidation coking and cracking coking, and it produces a large number of coke components with low hydrogen-to-carbon ratios, as well as lighter components; coke is the main fuel source for continuous in situ combustion. Research has shown that transition metal oxides can reduce the onset temperature of heavy oil oxidation, increasing gas production during heavy oil cracking, as well as the yield of light components [16,17,18]. In addition, it can stabilize the leading edge of the fire line and reform heavy oil to improve the recovery rate from in situ combustion.

Transition metals are widely used as catalysts in the refining of petroleum and its derivatives [19,20,21]. From the perspective of the technological process, the in situ combustion of crude oil can also be considered as a process for upgrading crude oil within the geological formation. Research on the use of transition metal oxides to catalyze high-temperature reactions in crude oil has been carried out for many years [22,23,24,25], but most research remains at the laboratory stage and is difficult to apply in oil fields. The catalyst cannot be completely and rapidly dispersed in heavy oil in the geological formation, which results in low catalytic efficiency and an unstable combustion leading edge during in situ combustion [26]. The catalyst size does not match the geological formation pore throat and can easily block the geological formation pores [27].

Recently, researchers synthesized efficient nanocatalysts with CeO_2_-doped Ni/Pd [28] and Pd and Au bimetals [29]. The study focused on the asphaltene-adsorption abilities of the catalysts and investigated the catalytic effects on asphaltenes under different conditions; experiments included differential scanning calorimetry (DSC) and high-pressure thermogravimetric analysis (HP-TGA) in a vacuum. The results showed that the synthesized catalysts have high catalytic efficiencies in the oxidation of C-7 asphaltenes. However, this catalytic system still failed to consider the injection capacity. This is because the catalysts for oil in situ combustion usually enter the geological formation by pre injection during application, and then the crude oil is ignited with the injected high temperature air. After the catalyst acts, the fire line in the geological formation moves, and the surface of the crude oil to be burned is covered with the catalyst. Therefore, when designing the catalyst structure, we pay particular attention to enabling the catalyst to reach the surface of the crude oil more efficiently in the complex geological formation environment.

In this study, a surface-active hyperbranched polymer was used as an encapsulation agent for platinum metal nanoparticles. This is a new type of heavy oil cracking catalyst, and could play a major role in heavy oil cracking. The synthesized catalyst would be prepared as a solution for injection before crude oil in situ combustion. This solution could carry highly efficient platinum nanoparticles to the surface of the crude oil, which would exhibit interfacial activity endowed by the hyperbranched polymer shell. The catalyst promotes the dehydrogenation and cracking of crude oil during in situ combustion. The crude oil could results in more combustion components after cracking, rather than their being lost in the form of light components, which would maintain the fire line in the geological formation. Platinum was selected as the nanometal core, and was prepared to yield a particle size less than 10.0 nm to increase the catalytic efficiency of the metal particles. The encapsulated metal nanoparticles were dispersed in an aqueous solution to form a nanometal fluid, with both surface activity and catalytic activity. At the same time, the hyperbranched polymer assembled from small molecules can interact with nanoparticles to catalyze heavy oil cracking, which further improves the ability of the catalyst to upgrade heavy oil. A nano-platinum metal fluid encapsulated by cetyl-hyperbranched poly(amide-amine) (CPAMAM) was synthesized and characterized, and the catalytic effect of the nanometal fluid on thermal cracking of Bohai heavy oil was studied.

## 2. Results

In this work, a hydrophilic hyperbranched polymer (HPAMAM) was synthesized from diethylenetriamine and methyl acrylate. A surface-active hyperbranched polymer, namely CPAMAM, was obtained by lipophilic modification with palmitoyl chloride. Characterization of the synthesized nanometal fluid revealed that the average size of the Pt particles was less than 6.0 nm in solution, and they could increase the catalytic efficiency in heavy oil cracking. The hyperbranched polymer shell modified by palmitoyl chloride showed better interfacial activity, which reduced the interfacial tension between the heavy oil and water to 1.97 mN/m. Thermal analysis of the heavy oil showed that adding 0.1 wt% nanometal fluid to the heavy oil improved the conversion rate by 4.7% during pyrolysis at 350 °C in a nitrogen atmosphere. The results of fitting to the Arrhenius equation showed that the activation energy of heavy oil cracking with 0.1 wt% nanometal fluid was reduced from 116.04 kJ/mol to 104.35 kJ/mol in a nitrogen atmosphere; the catalytic efficiency was better than that of both copper stearate and iron powder.

### 2.1. Structural Analysis of Hyperbranched Polymers

**Figure 1:**
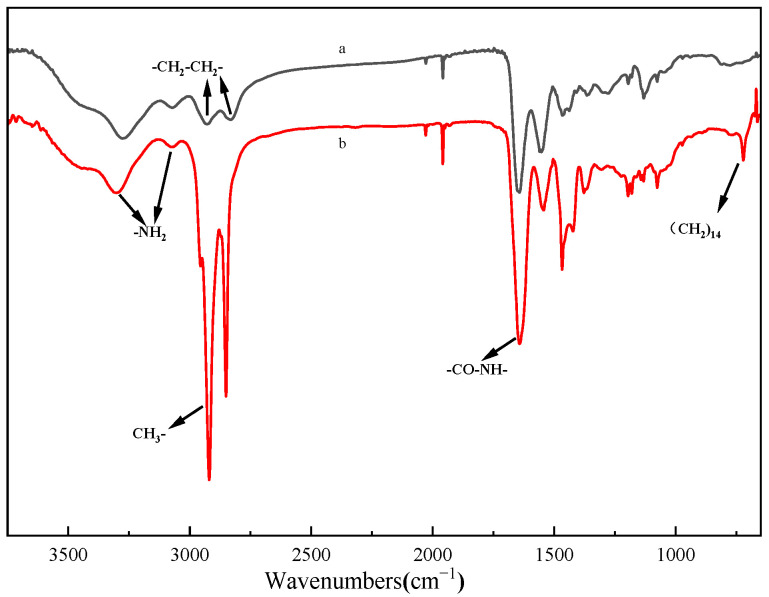
Fourier-transform infrared spectra of HPAMAM and CPAMAM. (a) HPAMAM and (b) CPAMAM.

The Fourier transform infrared spectroscopy (FTIR) spectra of HPAMAM and CPAMAM are shown in Figure 1. The –NH_2_ relative peak area at 3390 cm^−1^ in the HPAMAM spectrum is significantly lower after modification; this is because palmitoyl chloride consumes the terminal amino groups by a grafting reaction. In the FTIR spectrum of CPAMAM, the –CH_3_ and –CH_2_ peaks at 2800–3000 cm^−1^ are significantly enhanced, the typical (CH_2_)_14_ peak is clearly observed at 640 cm^−1^ [30], and an increased peak from –CO–NH– is observed at 1650 cm^−1^ [31]. Analysis of the IR spectra indicates that CPAMAM obtained an amide bond by a grafting reaction.

**Figure 2:**
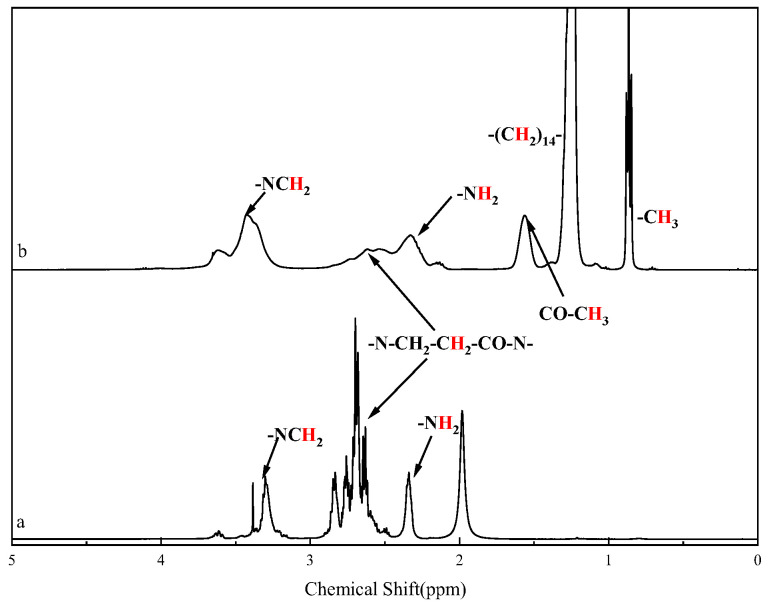
^1^H nuclear magnetic resonance spectra of HPAMAM and CPAMAM. (a) HPAMAM and (b) CPAMAM.

Figure 2 shows the ^1^H nuclear magnetic resonance (NMR) spectra of HPAMAM and CPAMAM. The signal from the cetyl groups of CPAMAM appears at 0.8–1.7 ppm. The signal from the methylene group linked to the amide bond shifts to 3.2–3.7 ppm, and the –NH_2_ signal at 2.3 ppm in the HPAMAM spectrum is weaker than that in the CPAMAM spectrum. The signals in the ^1^H NRM and FTIR spectra match those in the previously reported spectra of polymers [32].

The elemental and amine group contents of HPAMAM and CPAMAM are shown in Table 1. The nitrogen content of HPAMAM is close to the theoretical value, but the relative content of nitrogen in CPAMAM decreases because of the grafting of hydrocarbon chains. The changes in the total amine and tertiary amine contents of the polymers are consistent with the calculated nitrogen element content. Based on the changes in amine numbers, it can be inferred that the number of long carbon chain branches in each CPAMAM molecule accounts for approximately 25% of the grafting sites.

### 2.2. Structural Analysis of Nanometal Dispersions

**Figure 3:**
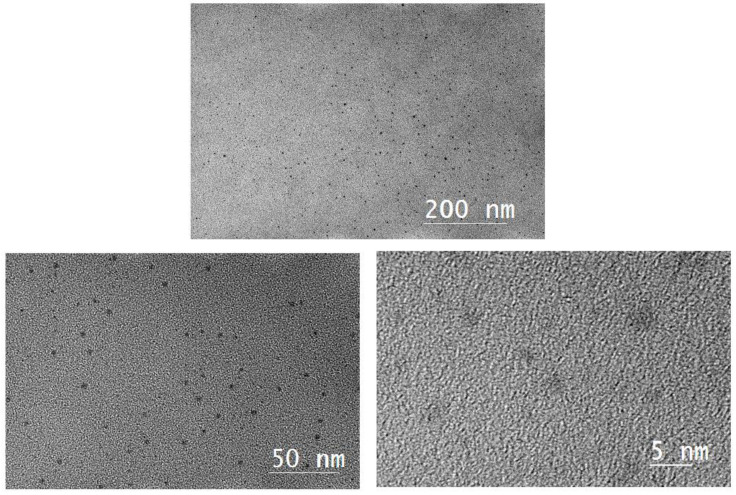
High-resolution transmission electron microscopy images of Pt@CPAMAM.

**Figure 4:**
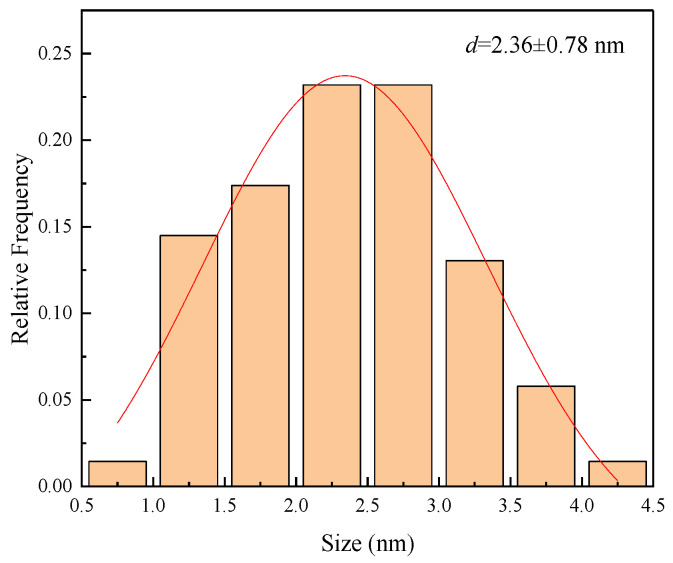
Size distribution in 0.1 wt% Pt@CPAMAM solution. Red line is result of Gauss fit for the test data.

High resolution transmission electron microscopy (HRTEM) images of the nanometal dispersions are shown in Figure 3 and Figure 4, revealing the size distribution of the metal particles calculated from the HRTEM images. Toluene was used as the dispersant for the nanometal. As the HRTEM images show, the metal particles are uniform in size and well dispersed, which indicates that the metal particles have good oil-phase dispersion abilities. The particle size range of the nanoparticles is 1.5–3.0 nm, which indicates that hyperbranched polymers were involved in regulating the growth of metal particles. The small particle size and excellent dispersion also increase the density of metal catalytic sites during in situ combustion.

**Figure 5:**
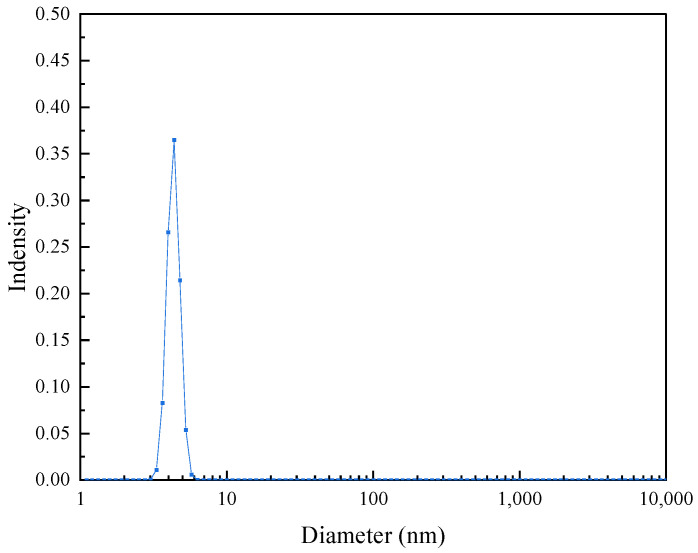
Size distributions of 0.1 wt% Pt@CPAMAM solution.

Figure 5 shows the average particle size of the nanometal fluid solution. In a 0.1 wt% solution, the average size of the dispersed particles is approximately 6.0 nm, which is the size occupied by hyperbranched polymer molecules in the solution. Although this size is larger than that of the nanometal particles, it is still one order of magnitude lower than the micron-scale pore throat in a geological formation; therefore, they can pass through the pore throats in geological formations smoothly.

**Figure 6:**
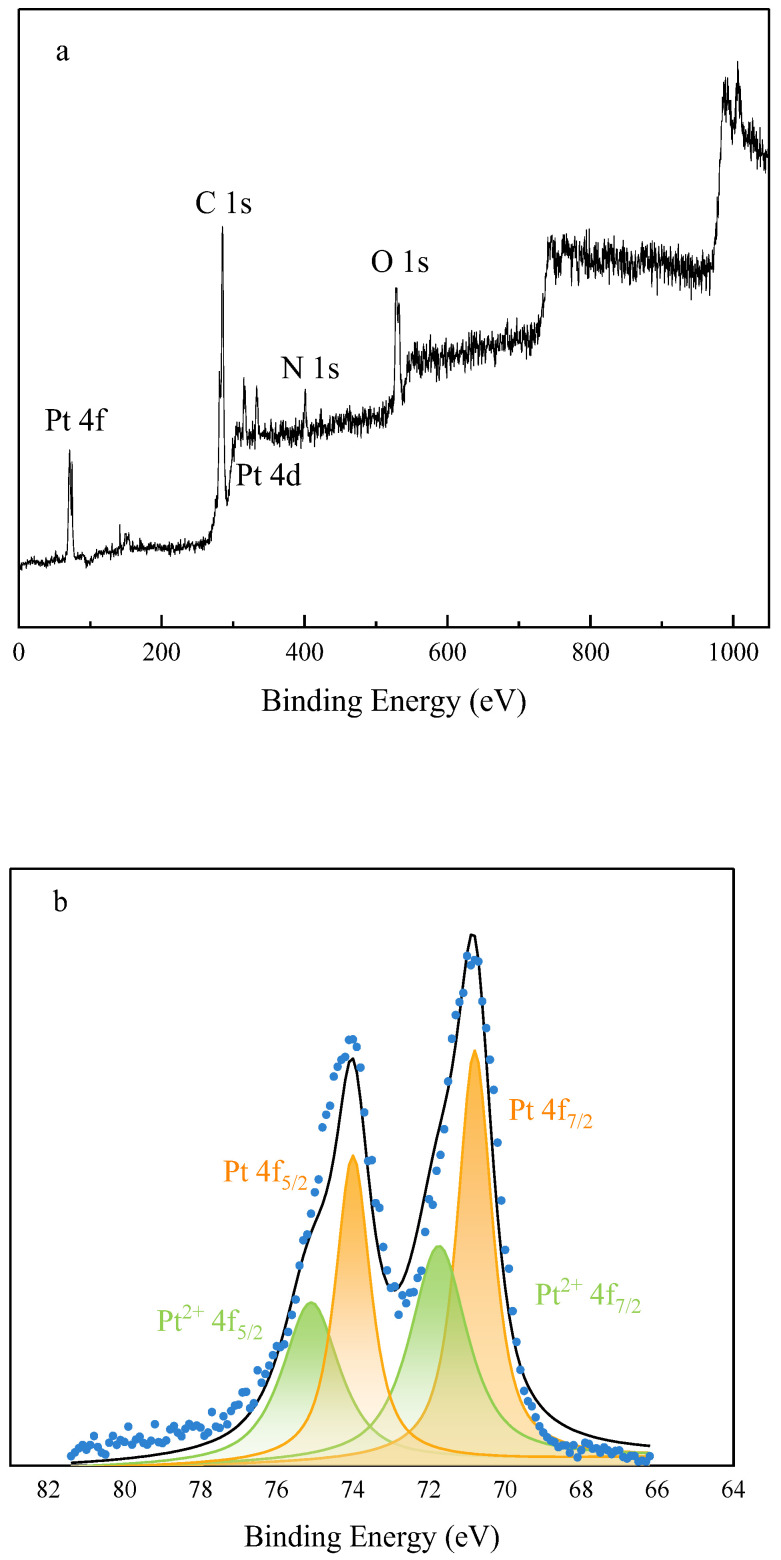

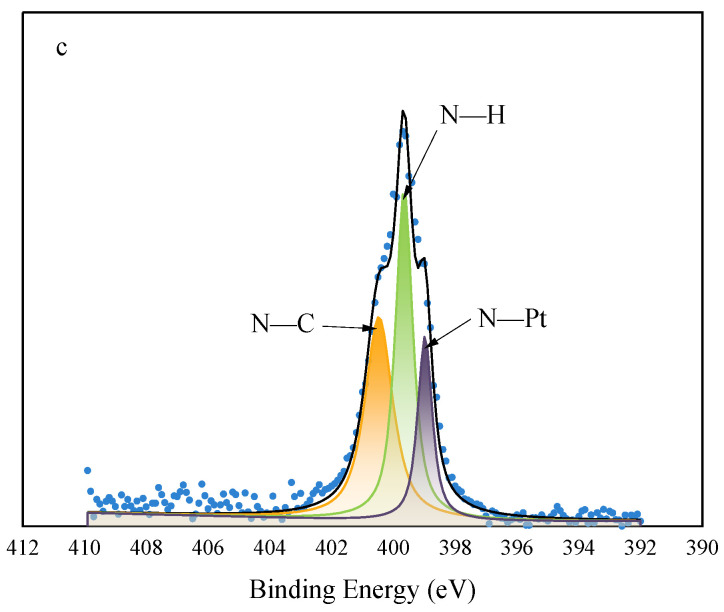
High-resolution X-ray photoelectron spectra of Pt@CPAMAM sample. (**a**) Full spectrum of Pt@CPAMAM sample, (**b**) Pt 4f region of Pt@CPAMAM sample, and (**c**) N 1s region of Pt@CPAMAM sample.

Figure 6a shows the full X-ray photoelectron (XP) spectrum of the Pt@CPAMAM sample; the signals for carbon, nitrogen, oxygen, and platinum elements are present in the spectrum. Figure 6b shows the high-resolution scan of the 4f orbital region of Pt and its peak-fitting results. The distinct double peaks arise from the spin–orbit splitting of Pt 4f_7/2_ and Pt 4f_5/2_, with binding energies of 70.9 eV (4f_7/2_) and 74.1 eV (4f_5/2_) eV. This indicates that the main forms of the platinum element are Pt(0) and Pt^2+^. Peak fitting of the integral peak areas shows that the contents of Pt(0) and Pt^2+^ in the sample are 63% and 37%, respectively. This indicates that the CPAMAM ligands modify and stabilize Pt nanoparticles by binding nitrogen atoms to the surfaces of the Pt atoms in the Pt nanoparticles; this is similar to the peak migration phenomenon of amine-coated Pt nanoparticles [33]. Figure 6c shows the N 1s XP spectrum of Pt@CPAMAM. Three peaks were observed near 400 eV after peak fitting. The N peak at 399.8 eV showed negative and positive shifts. The negative shifts clearly indicate electron transfer between N and Pt, proving that Pt in the sample binds to the polymer via coordinate bonds.

### 2.3. Thermal Properties of Polymers and Pt@CPAMAM

**Figure 7:**
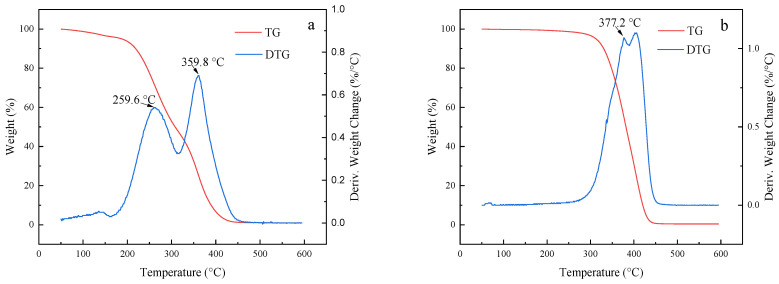

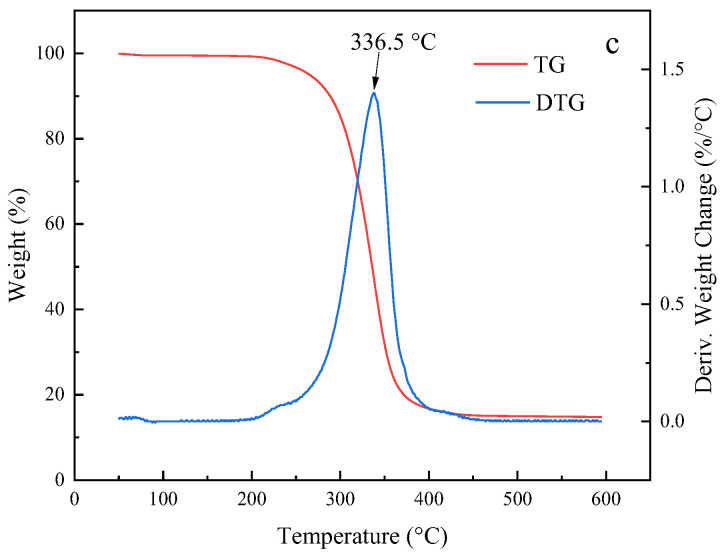
Thermogravimetric and differential thermogravimetric curves for HPAMAM/CPAMAM and Pt@CPAMAM nanoparticles. (**a**) HPAMAM, (**b**) CPAMAM, and (**c**) Pt@CPAMAM.

Figure 7 shows the thermogravimetric (TG) and differential thermogravimetric (DTG) curves for the two polymers and Pt@CPAMAM; the curves were obtained by using TGA. The thermogravimetric curve shows that the mass retention of the polymer decreased with increasing temperature, and the polymer was completely decomposed when the temperature exceeded 500 °C. The thermogravimetric curve for Pt@CPAMAM indicates that the Pt monomer content was as high as 19 wt%. A comparison of the thermogravimetric curves for CPAMAM with those of the nanometal dispersions reveals two peaks for the thermal decomposition of CPAMAM; the lower-temperature peak corresponds to a break in the long carbon chain, and the higher-temperature peak corresponds to decomposition of the polymer core. In the presence of the nanometal, the temperature for complete decomposition of the polymer decreased, and the weight-loss peak shifted to a lower temperature, which suggests that nanometals effectively catalyze the cracking of hydrocarbons at high temperatures.

### 2.4. Comparison of Interfacial Activities of HPAMAM and CPAMAM

**Figure 8:**
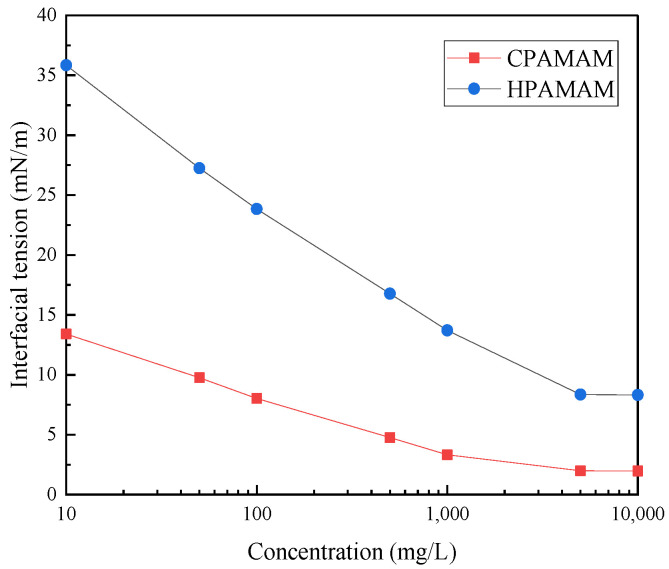
Interfacial tensions of HPAMAM and CPAMAM solutions of different concentrations.

Figure 8 shows the oil–water interfacial tensions for different concentrations of HPAMAM and CPAMAM. The interfacial activity of the modified polymer is better than that of HPAMAM. At the same mass fraction, the direct interfacial tension between heavy oil and water was reduced from 8.32 mN/m to 1.97 mN/m by modification with palmitoyl chloride, which indicates that the nanometal fluid diffuses more rapidly at the oil–water interface.

As shown in Table 2, the interfacial tension of the modified 1000 mg/L concentration of the hyperbranched polymer solution was slightly higher in low-concentration sodium chloride solution. Even when the sodium chloride concentration reached 17,500 mg/L, the interfacial tension of the polymer solution was still below 5 mN/m, which indicates that the formed nanometal solution exhibits salt resistance.

### 2.5. Catalytic Cracking of Heavy Oil with Pt@CPAMAM

**Figure 9:**
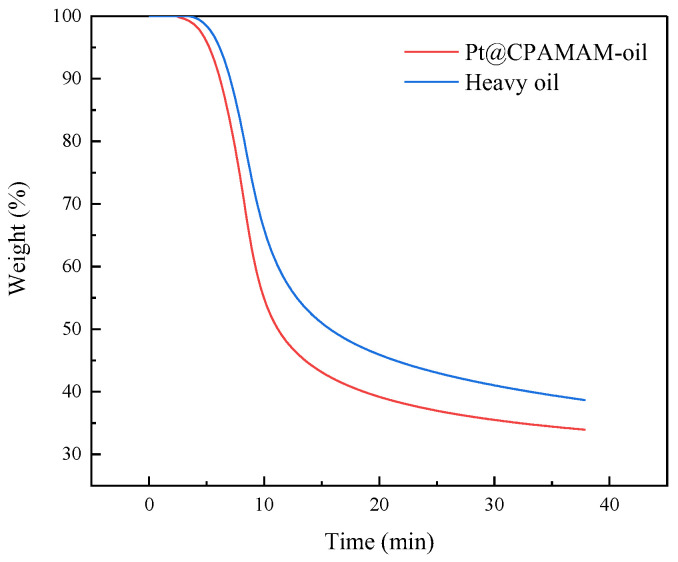
Effects of catalyst on isothermal cracking of Bohai heavy oil at 350 °C.

Figure 9 shows a comparison of the rates of cracking of heavy oil with 0.1 wt% Pt@CPAMAM after rapid heating at 100 °C/min to 350 °C and at constant temperature. The heavy oil containing the nanometal decomposed faster during the initial stage of decomposition, and the final residual mass decreased from 38.67% to 33.94%, which improves the conversion rate by approximately 5%. The heavy oil added to the nanometal fluids underwent a deeper cracking reaction at the same temperature, and the catalyst promoted the cracking of heavy oil under high-temperature conditions.

**Figure 10:**
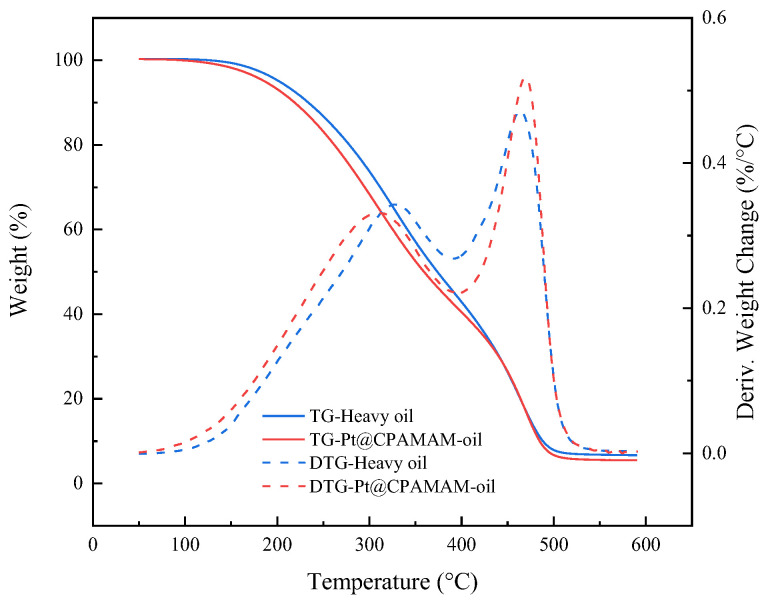
Thermogravimetric and differential thermogravimetric curves for heavy oil and Pt@CPAMAM-oil.

Figure 10 shows the weight losses for heavy oil and a Pt@CPAMAM-oil sample upon heating at 10 °C/min in a nitrogen atmosphere. The Pt@CPAMAM-oil sample contained 0.1 wt% nanometal fluid. The TG curve for Pt@CPAMAM-oil shows a lower temperature shift compared with that of the original sample, which indicates that the nanometal fluid added to the heavy oil reduced the decomposition temperature and accelerated decomposition of the heavy oil. Because the main function of the hyperbranched polymer shell is to disperse the nanometal particles and use its interfacial activity to carry the nanometal particles to the oil–water interface, in the oil in situ combustion process, the catalyst should be used before heating the geological formation to 350 °C. Before the crude oil starts to burn, the catalyst can contact the surface of the crude oil and catalyze oil cracking during combustion. Therefore, when the nanometal catalyst is injected, the decomposition of the hyperbranched shell does not influence the catalytic effect.

**Figure 11:**
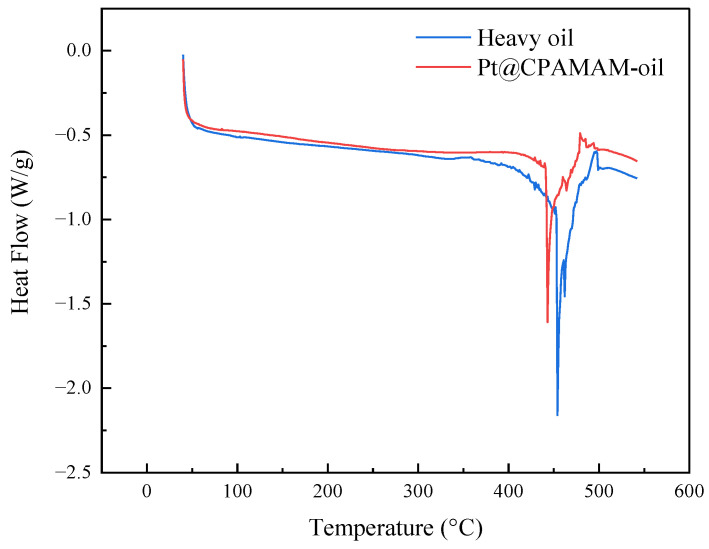
High-pressure differential scanning calorimetry curves for heavy oil and Pt@CPAMAM-oil.

Figure 11 shows the high-pressure differential scanning calorimetry (PDSC) curves for the heavy oil and the Pt@CPAMAM-oil sample. In a nitrogen atmosphere, the decomposition of heavy oil showed two distinct stages, and the heavy oil clearly showed heat absorption at 450 °C. There was also an obvious peak in the TGA curve for the heavy oil in the same temperature range.

We also investigated the changes in the carbon and hydrogen element contents after heavy oil cracking in a nitrogen atmosphere. A constant-volume device was used to heat the heavy oil to 350 °C in a nitrogen atmosphere for 20 min, with subsequent natural cooling. The catalyst used for cracking the heavy oil was 0.1 wt% Pt@CPAMAM; the results are shown in Table 3.

Table 3 shows the content of hydrocarbon elements in the solid-phase residue of crude oil after high-temperature cracking in a constant-volume device. The saturated hydrocarbon components in the crude oil undergo chain breaking, cyclization, and dehydrogenation reactions in this reaction system [34]. Aromatic hydrocarbon components condense to generate deposits with a high hydrocarbon ratio [35]. The results in Table 4 show that the hydrogen:carbon ratio of the deposits after adding a catalyst is lower, which indicates that the crude oil with a catalyst undergoes a deeper cracking reaction under the same conditions.

When the heavy oil in a geological formation is burned, the oil in contact with oxygen in the fire burns. The remaining part of the oil is heated to a high temperature and first cracked and then burned as the fire line advances. Heavy oil cracking is an endothermic chemical reaction, and the absorption heat is released in the subsequent combustion reaction according to the conservation of energy. The hydrogen content of the heavy oil with an added catalyst is lower after cracking because of deepening of the cracking reaction. This means that the heavy oil absorbs more heat at this stage, and the products release more heat when burned, which maintains the advance of the fire line.

### 2.6. Kinetic Analysis

According to the classical Arrhenius kinetic equation, a linear fitting relationship between the reaction activation energy and conversion can be obtained for the cracking reaction of heavy oil [36].

(1) $$\log\frac{dW/dt}{W}=\log A_{r}-\frac{E}{2.303RT}$$

where *dW*/*dt* is the mass loss rate, *W* is the remaining mass of the sample, *A*_r_ is the frequency factor, *E* is the activation energy, *R* is the universal gas constant, and *T* is the temperature. The frequency factor and activation energy can be determined by plotting the linear fitting line of log(*dW*/*dt*) versus 1/T. The kinetic parameters were obtained from TGA curves at heating rates of 2.5 °C/min, 5 °C/min, 10 °C/min, and 20 °C/min.

**Figure 12:**
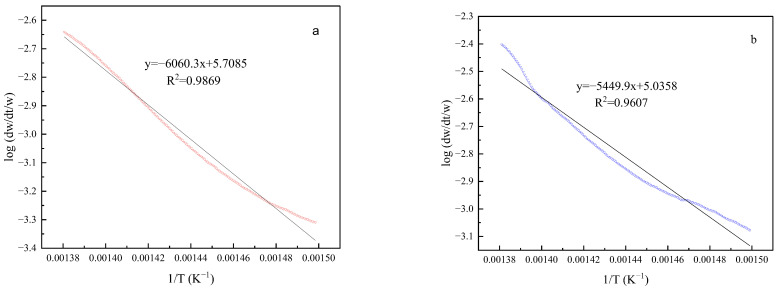
Kinetic parameter calculations for heavy oil and Pt@CPAMAM-oil by the Arrhenius procedure: (**a**) heavy oil and (**b**) 0.1 wt% Pt@CPAMAM-oil.

The activation energies of the heavy oil and the Pt@CPAMAM-oil sample were obtained by using the Arrhenius procedure, based on the TGA curves. Figure 12 shows the linear fitting curves for the two samples in the temperature range of 400–450 °C; the results are summarized in Table 4. Fitting of the TGA curves with the Arrhenius plots yielded correlation coefficients greater than 0.96, which indicates that the calculated kinetic parameters are highly accurate. Upon the addition of the nanometal fluid, the activation energy of the crude oil cracking reaction decreased from 116.04 kJ/mol to 104.35 kJ/mol, and because heavy oil containing the nanometal fluid was easier to crack in this temperature range, a higher conversion rate was achieved during thermal decomposition.

In addition, the effect of transition metal catalysts on the cracking of Bohai heavy oil was compared with the effect on Pt@CPAMAM cracking under the same conditions. Copper stearate and iron powder were selected as the catalysts for the comparison experiments. The results of the calculations of the kinetic parameters for heavy oil cracking with transition metal catalysts are shown in Table 5.

As shown in Table 5, copper stearate and iron powder were used as heavy oil cracking catalysts. When 0.1 wt% catalyst was added, the platinum nanoparticles synthesized in this work more obviously affected the reduction of the activation energy of the heavy oil cracking reaction. The synthesized catalyst solution exhibits surface activity, whereas copper stearate or iron powder do not.

## 3. Materials and Methods

### 3.1. Materials

Methanol (99.5%), anhydrous ether (99.7%), triethylamine (99.0%), chloroform (99.0%), methyl acrylate (MA, 99%), palmitoyl chloride (98%), chloroplatinic acid hexahydrate (H_2_PtCl_6_·6H_2_O, AR, Pt ≥ 37.5%), tetraoctyl ammonium bromide (TOAB, 98%), sodium borohydride (98%), copper stearate (98%), and iron powder (99.9%, 100 nm) were purchased from Shanghai Aladdin Biochemical Technology Co., Ltd. (Shanghai, China) Dichloromethane (99.5%), diethylenetriamine (DETA, 99%), and toluene (99.5%) were obtained from Sinopharm Chemical Reagent Co., Ltd., Shanghai, China (Shanghai All of the reagents were used as received, without further purification. The Bohai heavy oil was obtained from the JXI-1 oilfield, and the basic properties and SARA fractions (saturates, aromatics, resins, and asphaltenes) of this heavy oil are listed in Table 6; the SARA fractions were tested by chromatographic column separation, and the adsorption material is 200 mesh alumina powder.

### 3.2. Synthesis of Hyperbranched Nanometal Fluids

#### 3.2.1. Synthesis of HPAMAM

The typical HPAMAM synthesis procedure is described as follows. DETA (20.63 g) was dissolved in 25.5 mL methanol, 21.6 mL MA was slowly added, and the mixture solution was stirred at 35 °C for 48 h. The mixture was distilled under vacuum, and the temperature was increased from 60 °C to 140 °C, as follows: 1 h at 60 °C, 1 h at 80 °C, 1.5 h at 100 °C, 1.5 h at 120 °C, and 1.5 h at 140 °C; this process resulted in a yellow-green viscous liquid. The product was dissolved in 50 mL methanol, then poured into 300 mL anhydrous ether and stirred for 30 min. The upper layer of ether was separated after standing, and an additional 200 mL of anhydrous ether was added twice for washing. The layered product was vacuum dried at 60 °C for 4 h, and the final yellow-green viscous product was HPAMAM.

#### 3.2.2. Modification of HPAMAM

The HPAMAM synthesized in the previous step (1.0 g with 0.011 mol primary and secondary amino groups) was dissolved in 12 mL chloroform, 1.13 g triethylamine was added as an acid-acceptor, and the mixture was transferred to a flask. Palmitoyl chloride (1.0 g) was dissolved in 6 mL chloroform, and this palmitoyl chloride solution was added into a flask dropwise under an anhydrous environment. The mixture was stirred at 35 °C for 24 h and washed twice with anhydrous ether. The precipitate was dried in a vacuum at 60 °C for 4 h to produce a pale yellow waxy solid substance called cetyl-hyperbranched poly(amide-amine) (CPAMAM).

### 3.3. Synthesis of Nanometal Fluids

**Figure 13:**
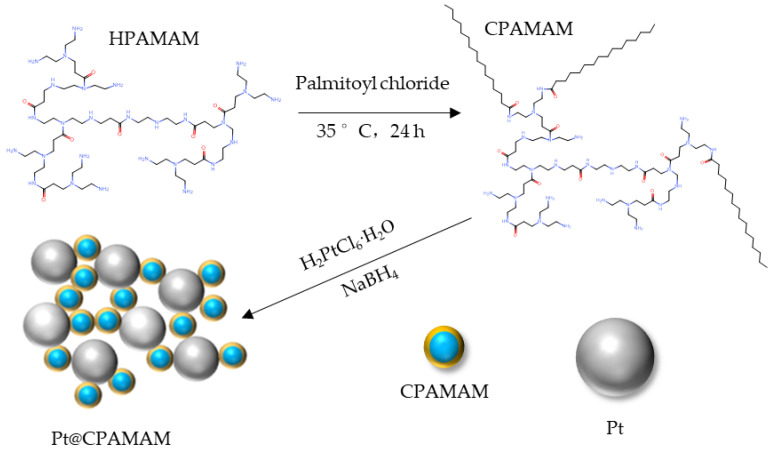
Schematic of the synthesis of Pt nanoparticles fluids by CPAMAM.

The strategy for synthesizing Pt@CPAMAM nanoparticles is schematically shown in Figure 13. In a typical process, a solution of 0.2 g H_2_PtCl_6_·6H_2_O in water was mixed with a solution of 0.5 g TOAB in toluene. The mixture was stirred for 30 min, separated, and the aqueous phase was removed. After dissolving 0.4 g CPAMAM into the system, NaBH_4_ (0.15 g in water) was added dropwise to the solution under stirring. The synthesis temperature was 50 °C. After 6 h, the synthesized Pt@CPAMAM nanoparticles were separated by rotary evaporation, washed with ether, centrifuged, and dried under vacuum.

### 3.4. Characterization of CPAMAM and Pt@CPAMAM Nanoparticles

The structure of hyperbranched poly(amide-amine) was characterized by nuclear magnetic resonance (NMR) and Fourier transform infrared spectroscopy (FTIR). The hydrogen spectrum was recorded on an AVANCE III 400 MHz spectrometer (Bruker, Zurich, Swiss) in deuterated methanol solvent. The FTIR spectra were recorded on a Nicolet iS10 spectrometer (Thermo Fisher Scientific, Waltham, MA, USA). The element contents of hydrogen and carbon and nitrogen in HPAMAM and CPAMAM were analyzed by elemental analysis (EA) on a Vario Micro element analyzer (Elementar Analysensysteme GmbH, Frankfurt, German). The thermal properties of HPAMAM and Pt@CPAMAM were tested by thermogravimetric analysis (TGA) under a nitrogen atmosphere on a Q50 analyzer (TA Instruments, New Castle, DE, USA). The heating rate was set at 10 °C/min and the scanning temperature range was 100–600 °C. The content of the primary amine and secondary amine groups in HPAMAM was calculated by measuring the total amine values and the content of tertiary amine groups by direct titration; the detailed experimental methods are shown in SM1 and SM2 of the Supplementary Materials.

### 3.5. Characterization of Nanometal Fluids

The morphology and size distribution of Pt@CPAMAM were characterized by high resolution transmission electron microscopy (HRTEM) images on an JEM2100F microscope (JEOL, Tokyo, Japan), with an accelerating voltage of 200 kV. The surface chemical state of Pt@CPAMAM was obtained by X-ray photoelectron spectroscopy (XPS) measurements on an Escalab 250Xi instrument (Thermo Fisher Scientific, Waltham, MA, USA). The thermal properties of Pt@CPAMAM samples were tested under nitrogen atmosphere on a Q50 analyzer (TA Instruments, New Castle, DE, USA). The heating rate was set at 10 °C/min, and the scanning temperature range was 50–800 °C. The average particle size of Pt@CPAMAM in solution was characterized by a Nano ZS dynamic light scattering instrument (Malvern, Malvern, UK).

### 3.6. Performance Evaluation of Nanofluids

Nanometal fluids of different mass fractions were prepared, and the surface tension and oil–water interfacial tension of different concentrations of fluids were tested by a spinning drop interfacial tensiometer TX-500C (Shanghai Geology Instrument Institute, Shanghai, China). All of the interfacial tension measurements were conducted at 25 °C under atmospheric pressure. The thermal catalytic cracking effect of nanometal fluids on Bohai heavy oil at different heating rates was carried out using the Q50 thermogravimetric analyzer (TGA, TA Instruments, New Castle, DE, USA), under a nitrogen atmosphere. A Q2000 differential scanning calorimeter (DSC, TA Instruments, New Castle, DE, USA) was applied to investigate the thermal behavior of the heavy oil. A EA3000 elemental analyzer (EuroVector, Pavia, Italy) was used to determine the content of carbon and hydrogen in the heavy oil.

## 4. Conclusions

In this work, hyperbranched poly(amide-amine) was used as the skeleton vector for nanometals. Pt nanoparticles were prepared in a dispersion solution with a surfactant, which enabled the migration of metal particles at the oil–water interface. Characterization of the synthesized nanometal fluid revealed that the average size of the Pt particles was less than 6.0 nm in solution, and this could increase the catalytic efficiency in heavy oil cracking. The hyperbranched polymer shell modified with palmitoyl chloride exhibited better interfacial activity, which reduced the interfacial tension between the heavy oil and water to 1.97 mN/m. The excellent interfacial activity ensured migration of the nanometal fluid in the geological formation. The application of a heavy oil combustion catalyst always occurs in a porous medium, and the flow ability of nanometal fluid is not only decided by its size, but also the interfacial tension of the fluid. This work differs from previous research in the overall structural design of the catalyst, which provides a novel synthesis approach for catalysts used in heavy oil in situ combustion. However, the effect of nanometal fluid on the combustion of heavy oil in a geological formation requires further study.

## Figures and Tables

**Table 1:** Elemental and amine group contents of HPAMAM and CPAMAM.

Sample Name	Carbon Content (wt%)	Hydrogen Content (wt%)	Nitrogen Content (wt%)	Total Amine Content (mol/g)	Tertiary Amine Content (mol/g)
HPAMAM	50.07	9.26	22.17	0.016	0.005
CPAMAM	55.85	10.12	18.43	0.013	0.005

**Table 2:** Oil–water interfacial tension of 1000 mg/L concentration of CPAMAM in sodium chloride solution at different concentrations.

Concentration, mg/L	Interfacial Tension, mN/m
0	3.33
3500	2.48
7000	2.33
10,500	2.88
14,000	3.29
17,500	3.55

**Table 3:** Comparison of carbon and hydrogen contents of heavy oil and Pt@CPAMAM-oil.

Sample Name	C (wt%)	H (wt%)
Heavy oil	89.282	11.708
Cracked oil	96.771	3.329
Cracked Pt@CPAMAM-oil	97.453	2.447

**Table 4:** Kinetic parameters of heavy crude oil and Pt@CPAMAM-oil, calculated by using the Arrhenius procedure.

Sample	E (kJ/mol)	A_r_ (1/min)	R^2^
Oil	116.04	5.111 × 10^5^	0.9869
Pt@CPAMAM-Oil	104.35	1.086 × 10^5^	0.9607

**Table 5:** Comparison of activation energies with different catalysts.

Sample	E (kJ/mol)	A_r_ (1/min)	R^2^
Heavy oil	116.04	5.111 × 10^5^	0.9869
Copper stearate–oil	115.4	9.06 × 10^5^	0.9841
Iron powder–oil	108.5	2.61 × 10^5^	0.965
Pt@CPAMAM–oil	104.35	1.086 × 10^5^	0.9607

**Table 6:** Basic properties and SARA fractions of Bohai heavy oil.

Density (g/cm^3^@25 °C)	Viscosity (mPa·s@25 °C)	SARA Composition (wt%)			
		Saturates	Aromatics	Resins	Asphaltenes
1.0066	20,610	33.31	18.90	36.25	11.54

## Data Availability

Data is contained within the article. The data present in this study are available in article.

## Supplementary Materials

The following supporting information can be downloaded at: https://www.mdpi.com/article/10.3390/molecules28145328/s1, SM1. Determination of the total amine values in HPAMAM; SM2. Determination of the content of tertiary amine groups in HPAMAM.
