# Supplementary material for: Preparation of Surface-Active Hyperbranched-Polymer-Encapsulated Nanometal as a Highly Efficient Cracking Catalyst for In Situ Combustion of Heavy Oil
Source: Molecules. 2023 Jul 11;28(14):5328. doi: 10.3390/molecules28145328 (PMC10383047; doi:10.3390/molecules28145328)
Supplement: Supplementary file 1 [file molecules-28-05328-s001.zip › molecules-2423049-supplementary.pdf]

Preparation of surface active hyperbranched polymers encapsulated nanometals as a highly efficient cracking catalysts for in-situ combustion of heavy oil

Ao Sun[12], Chenyang Yao[2], Lifeng Zhang[3], Yanmin Sun[3], Jun Nan[3], Houkai Teng[3], Jiazhong Zang[3], Lishan Zhou[3], Zhenzhong Fan[1], Qilei Tong[1*]

[1] School of Petroleum Engineering, Northeast Petroleum University, Daqing, China
2 Department of Chemistry, Zhejiang University, Hangzhou, China
3 CenerTech Tianjin Chemical Research & Design Institute Co., Ltd, Tianjin 300131, China
* Corresponding author. Key Laboratory of Improving Oil Recovery by Ministry of Education, Northeast Petroleum University, Daqing, China

SM1. Determination of the total amine values in HPAMAM

The total amine values of HPAMAM was determined by the direct titration with hydrochloric acid-ethanol (the concentration of HCl was 0.1 mol/L). Typically, 0.1 g HPAMAM was added into a conical flask with 40 mL anhydrous ethanol and dissolved completely. Then two drops of 0.1% bromophenol blue ethanol (0.1g bromophenol blue was dissolved in 100 mL ethanol) was added as the indicator. The titration was conducted by the hydrochloric acid-ethanol solution until the solution color changed into yellow, and the volume of the consumed hydrochloric acid-ethanol solution was recorded. The total amine value was calculated as following equation:

$$X=(\mathrm{Mr}_{KOH}\times C_1\times V_1)/m_1$$

where X was the total amine values (mol KOH/g); C_1 was the concentration of hydrochloric acid-ethanol solution (mol/L); V_1 was the consumed volume of hydrochloric acid-ethanol solution (mL); m_1 was the mass of HPAMAM(g).

SM2. Determination of the content of tertiary amine groups in HPAMAM

The content of tertiary amine groups in HPAMAM was determined by the direct titration with perchloric acid-acetic acid (the concentration of perchloric acid was 0.1 mol/L). 0.2 g HPAMAM was added into flask with 20 mL acetic anhydride and 2 mL acetic acid. Heating reflux in the flask for 45 minutes, while adding glass drops to prevent bumping. Then rinse the condenser tube and flask with 50 mL of acetic acid and transfer to a 250 mL beaker. Two drops of gentian violet solution (0.5 g gentian violet was dissolved in 100 mL acetic acid) were added as the indicator. The titration was conducted by the perchloric acid-acetic acid solution until the solution color changed into blue, and the volume of the consumed perchloric acid-acetic acid solution was recorded. The the content of tertiary amine groups was calculated as following equation:

$Y=(\mathrm{Mr}_{KOH}\times C_2\times V_2)/m_2$

Where Y was the content of tertiary amine groups (mol KOH/g); C_2 was the concentration of perchloric acid-acetic acid solution (mol/L); V_2 was the consumed volume of perchloric acid-acetic acid solution (mL); m_2 was the mass of HPAMAM (g).

The content of primary amine and secondary amine groups was calculated as following equation:

$A=X-Y$

Where A was the content of primary amine and secondary amine groups.
